# BeQuIK (Biosensor Engineered Quorum Induced Killing): designer bacteria for destroying recalcitrant biofilms

**DOI:** 10.1111/1751-7915.13465

**Published:** 2019-07-21

**Authors:** Pinpunya Riangrungroj, Karen M. Polizzi

**Affiliations:** ^1^ Department of Life Sciences Imperial College London London SW7 2AZ UK; ^2^ Imperial College Centre for Synthetic Biology Imperial College London London SW7 2AZ UK; ^3^ Department of Chemical Engineering Imperial College London London SW7 2AZ UK

## Abstract

This opinion piece describes a new design for the remediation of recalcitrant biofilms. It builds on previous work to develop engineered *E. coli* that recognize quorum sensing signals from pathogens in a biofilm and secrete toxins in response. To solve the challenge of dilute signalling molecules, we propose to use nanobodies and enzymes displayed on the surface of the cells to localize them to the biofilm and degrade the extracellular polymeric substances, thus creating a solution with better ‘seek and destroy’ capabilities.

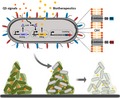

A bacterial biofilm is a cluster of cells residing within a self‐produced matrix of extracellular polymeric substance (EPS) containing primarily proteins, polysaccharides and extracellular‐DNA (Hoiby *et al*., 2015). Biofilm formation begins when planktonic cells attach to biotic or abiotic surfaces to form a microcolony, followed by EPS secretion. The biofilm matures into a three‐dimensional structure that is difficult to penetrate or degrade in a process governed by bacterial quorum sensing (QS) via signalling molecules in accordance with population density (Miller and Bassler, 2001). Ultimately, the cell cluster is dispersed to release planktonic cells back into the environment to continue the cycle (Jamal *et al*., 2018).

Bacterial biofilms negatively impact human health in two notable areas. In medical settings, they are associated with chronic infections of tissues and organs (e.g. cystic fibrosis and wounds) or implanted medical devices (e.g. catheters, endotracheal tubes, tissue fillers), where the biofilms effectively hide bacteria from the host immune system and render them up to 1000‐fold less susceptible to antibiotics than in their planktonic state (Gilbert *et al*., 2002). Attempts to treat biofilm infections with antibiotics negatively impact the environment due to their prolonged use and continuous discharge, since chronic exposure to antibiotics, even at sublethal concentrations, can promote a pool of resistance genes in natural bacterial communities (Sengupta *et al*., 2013, Andersson and Hughes, 2014). In food and drink manufacturing settings, bacterial biofilms can contaminate plant pipelines, resulting in production losses, economic damage and the potential for infections in consumers (Galie *et al*., 2018; Wang, 2019). However, not all biofilms are harmful; they can be useful for catalysing specific biotransformations and for bioremediation of toxic compounds (Benedetti *et al*., 2016).

Due to the emergence of antibiotic resistance around the globe, many alternative strategies have been developed to eradicate biofilm formation, including physical (heat and ultrasound) (Cai *et al*., 2017; Ricker and Nuxoll, 2017; Wang *et al*., 2018), chemical (organic acids) (Ryssel *et al*., 2009; Ban *et al*., 2012; Singla *et al*., 2014) and biological (bacteriophage) (Tkhilaishvili *et al*., 2018; Gupta *et al*., 2019) methods. However, there are some limitations to existing treatment methods. For example, the use of chemical disinfectants can trigger bacteria to activate acid tolerance responses or accumulate mutations to survive the low‐pH environment (Bearson *et al*., 1997; De Biase and Lund, 2015). Alternatively, the application of host‐specific bacteriophages is currently limited by a narrow host range, the emergence of phage resistance and phage inactivation by the human immune system (Donlan, 2009).

With the advent of synthetic biology, which aims to develop living organisms with genetically programmed responses, there has been wide interest in engineering commensal bacteria to address biofilms. Studies have primarily relied on the detection of QS molecules because they are reliable signals of biofilm formation and a wealth of genetic parts exist that can be used for engineering. Early work demonstrated the potential use of engineered *E. coli* to sense and kill *Pseudomonas aeruginosa*, by the production of protein toxins upon detection of QS autoinducers (Saeidi *et al*., 2011; Gupta *et al*., 2013). Despite the successful demonstration, the system relies on the diffusion of the toxins to the pathogen, making biofilm penetration a key issue. Later, Hwang *et al*. (2014) demonstrated improved targeting of *P. aeruginosa* by reprogramming the expression of the chemotaxis protein CheZ (phosphatase‐activating protein) to *cause E. coli* to swim towards the pathogen in response to secreted QS molecules. As a result, the engineered *E. coli* cells were able to kill both planktonic and biofilm‐residing cells. However, this system still relies on diffusion of the QS molecules, which may limit the distance over which it is effective.

An alternative way to localize engineered *E. coli* to biofilms would be via direct detection of the EPS components themselves. These large macromolecules would be difficult to detect via genetic regulators such as activators or repressors due to their slow diffusion and inability to cross the cell membrane. However, to facilitate adhesion of engineered *E. coli* cells to biofilms, one possible option could be the display of recombinant antibodies (Abs) or Ab fragments such as ‘nanobodies’ (Nbs) on the surface to physically bind the cells to the EPS. Nbs are single‐domain antibodies derived from the heavy chain Abs of camelids, which, unlike classical Abs, are devoid of the light chain and the CH1 constant domains (Deffar *et al*., 2009; Muyldermans, 2013). Nbs have emerged as next‐generation Abs for an array of diagnostic and therapeutic applications due to their small size (~15 kDa), high binding affinity, pH and thermal stabilities, hydrophilicity, ease of production in prokaryotic and eukaryotic hosts and low immunogenicity *in vivo* (Khodabakhsh *et al*., 2019; Salvador *et al*., 2019). A handful of Nbs against proteins involved in biofilm formation, e.g., biofilm‐associated protein (Bap) (Payandeh *et al*., 2014) and flagellin (Adams *et al*., 2014), have already been discovered, and it is feasible to imagine that Nbs against new targets could be selected from libraries (McMahon *et al*., 2017) or via immunization. Nbs against chemical targets have been selected before (Kim *et al*., 2012; Bever *et al*., 2016). Therefore, they are capable of binding to small molecules.

Salema *et al*. (2013) demonstrated *E. coli* surface display of Nbs by fusing them to the N‐terminal β‐intimin domain and an extracellular Ig‐like domain (D0) (Fig. 1). The intimin‐Nb fusion constructs have been successfully used to select specific Nbs against different targets (Salema *et al*., 2016a,a,b) and to serve as the detection element in whole‐cell biosensors (Kylilis *et al*., 2019). In addition, they can act as synthetic adhesins to specifically attach to surface antigens on target cells such as solid tumours (Pinero‐Lambea *et al*., 2015). Recently, Glass and Riedel‐Kruse (2018) demonstrated the use of the intimin‐Nb fusions to programme multicellular morphologies, which could be possibly further applied towards engineering synthetic cell consortia for microbiome therapy (Timmis *et al*., 2019). To date, there are no reports of surface display of enzymes via the β‐intimin domain (Salema *et al*., 2013), but other fusion partners have been previously used successfully in *E. coli* (Schuurmann *et al*., 2014; Qu *et al*., 2015; Zhang *et al*., 2018).

**Figure 1:**
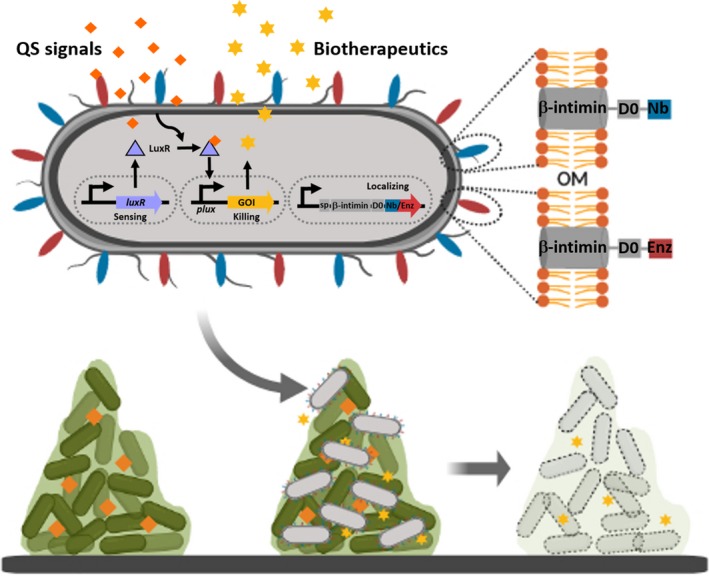
Design of QS‐dependent engineered *E. coli* via nanobody surface display for battling biofilm infections. The system consists of three modules: the surface co‐expressions of specific Nbs for pathogen identification and biofilm‐degrading enzymes (localizing), the constitutive expression of LuxR (sensing) for forming complex molecules with diffusible QS signals from pathogens and those complexes bind to *plux* promoter regulating the gene expression exhibiting antimicrobial and antibiofilm activities (killing). D0, extracellular Ig‐like domain; Enz, biofilm‐degrading enzyme; GOI, gene of interest; Nb, nanobody; OM, outer membrane; sp, signal peptide.

Here, we propose BeQuIK (Biosensor Engineered Quorum Induced Killing), a design for battling recalcitrant biofilms. BeQuIK is composed of three genetic modules introduced into *E. coli* cells in order to (i) target and degrade biofilms, (ii) sense the presence of pathogenic organisms and (iii) activate the production of toxins to kills these organisms. The novelty of BeQuIK lies in first module, which aims to solve the problems of previous ‘seek‐and‐destroy’ engineered cells by aiding in the targeting and penetration of biofilms. To achieve this, cells would display one or more biofilm‐targeting Nb (to recognize and bind to components of the EPS or biofilm‐mediated proteins) (Ardekani *et al*., 2013; Adams *et al*., 2014; Payandeh *et al*., 2014) and one or more biofilm‐degrading enzyme domains. The latter could consist of glucohydrolase enzymes, DNaseI, cellulase, etc. (Stiefel *et al*., 2016) and would allow for more effective diffusion of the QS signals needed to activate the killing mechanism as well as facilitating entry of the therapeutic agents meant to destroy the pathogenic bacteria. The proteins could either be displayed separately on the cell surface or as a fusion protein (Fig. 1).

Our design represents a new perspective for treating biofilm infections effectively by improving target localization based on physical linking between Nbs and specific antigens in the EPS. The engineered *E. coli* cells would also target planktonic cells for the prevention of biofilm formation leading to pathogen eradication. With accessible resources of Nbs and other materials, we envision that it is a feasible alternative for application to both biotic and abiotic surfaces, especially as a cleaning‐in‐place method for medical devices and industrial pipes. Furthermore, the sensing module and a responsive promoter in the killing module can be tailored to target any pathogens via its characteristic QS molecules for achieving an improved ‘seek‐and‐destroy’ system.

## Conflict of interest

None declared.
